# Draft Genome Sequence of *Haemophilus parasuis* gx033, a Serotype 4 Strain Isolated from the Swine Lower Respiratory Tract

**DOI:** 10.1128/genomeA.00224-13

**Published:** 2013-05-23

**Authors:** Jun Li, Hao Peng, Li-gan Xu, Yu-zhou Xie, Xiong-biao Xuan, Chun-xia Ma, Shuai Hu, Ze-xiang Chen, Wei Yang, Yong-ping Xie, Yan Pan, Li Tao

**Affiliations:** Department of Bacteriology, Guangxi Veterinary Research Institute, Nanning, Guangxi, Chinaa; Guangxi Key Laboratory of Animal Vaccines and Diagnostics, Nanning, Guangxi, Chinab; Guangxi University, Nanning, Guangxi, Chinac

## Abstract

*Haemophilus parasuis* serotype 4 is a Gram-negative pathogen that is the most prevalent *H. parasuis* serovar in the world, but its genome sequence information has not yet been reported. Thus, we determined the genome of *H. parasuis* strain gx033, a serovar 4 strain isolated from a lung specimen of a diseased piglet in southwestern China. Here, we present the first draft genome sequence of this species.

## GENOME ANNOUNCEMENT

*Haemophilus parasuis*, a Gram-negative NAD-dependent rod-shaped bacterium, is generally considered an important pathogen and the etiological agent of Glässer’s disease, which is characterized by fibrinous polyserositis, polyarthritis, and meningitis and causes significant financial loss worldwide (1). To date, 15 serovars have been described, using an immunodiffusion test. Serotypes 1, 2, 4, 5, 10, 12, 13, 14, and 15 are considered to be virulent and cause meningitis and pneumonia in swine (2). In North America, serotypes 4 and 5 are the most frequently isolated (3). By combining the results of both the gel diffusion (GD) and the indirect hemagglutination (IHA) test, serovars 4 (24.2%) and 5 (19.2%) were also determined to be the most prevalent serovars in China (4).

Though *H. parasuis* serotype 4 is a predominant pathogen in many countries, its genome sequence information has not been reported. Thus, we sequenced the genome of *H. parasuis* gx033, a serovar 4 strain isolated from a lung specimen of a diseased piglet in southwestern China. The genome sequence of strain gx033 was sequenced with a strategy involving the high-throughput Solexa paired-end sequencing technology (5), using Illumina HiSeq 2000 (Beijing Genomics Institute at Shenzhen, China) (6). A small (500-bp) library and a large (2,000-bp) library were constructed. Sequencing was performed with the pair-end strategy of 90-bp reads to produce 310 Mb of filtered sequences in the 500-bp library and 160 Mb in the 2,000-bp library. The reads were first assembled into 121 contigs with the SOAP*denovo* 1.05 (http://soap.genomics.org.cn/soapdenovo.html) (7). The contigs were then joined into 35 scaffolds using paired-end information. The genome sequence was analyzed using Glimmer 3.02 (8) for the protein-coding genes, tRNA-scan-SE (9) for tRNA, RNAmmer (10) for rRNA, RepeatMasker and RepeatProteinMasker for transposons, and Tandem Repeats Finder (http://tandem.bu.edu/trf/trf.html) for tandem repeat sequences. The functions of the predicted protein-coding genes were then annotated through comparisons with the Clusters of Orthologous Groups (COG) (11), KEGG (12), and NCBI-NR (13) databases.

The draft genome sequence analysis of gx033 showed a genome size of 2,155,493 bp, with a mean G+C content of 39.79%. In addition, 17,134-bp DNA transposons, 735-bp long interspersed repeated sequences, 2,098-bp long terminal repeated transposons, and 518 short interspersed repeated sequences were found. As is commonly found for other microbial genome sequences, 9.18% of the coding sequences (CDS) correspond to hypothetical proteins of unknown functions. In addition, 116 genes were predicted to encode proteins conferring cell wall, membrane, and envelope biogenesis and 46 genes were related to intracellular trafficking, secretion, and vesicular transport. Furthermore, eight genes related to cell motility were found, such as the gene *pilF* (GL000432), which encodes fimbrial biogenesis and twitching motility protein, which plays a vital role in the adherence to mucosal epithelia in mediating bacteria (14).

This is the first report of the genome sequence of *H. parasuis* serotype 4, and this information should provide further insight into the physiology and metabolic potential of the pathogenesis of the predominant *H. parasuis* serotype; the availability of this genome sequence will provide a better-defined genetic background for future studies of gene expression and regulation.

### Nucleotide sequence accession numbers.

This genome sequence has been deposited at DDBJ/EMBL/GenBank under the accession no. AOSU00000000. The version described in this paper is the first version, accession no. AOSU01000000.
